# Maxillary Central Incisor Eruption Failure Due to Supernumerary Teeth and Pericoronal Hamartoma: A Report of a Rare Case

**DOI:** 10.7759/cureus.64620

**Published:** 2024-07-15

**Authors:** Misa Ishiyama, Shunsuke Namaki, Hiroki Tamura, Shoko Ozawa, Takashi Kikuiri

**Affiliations:** 1 Department of Pediatric Dentistry, Nihon University School of Dentistry, Tokyo, JPN; 2 Department of Oral and Maxillofacial Surgery, Nihon University School of Dentistry, Tokyo, JPN

**Keywords:** supernumerary teeth, pericoronal hamartoma, maxillary central incisor, impaction of permanent teeth, eruption failure

## Abstract

Impaction of permanent teeth during the replacement period is a relatively common occurrence in clinical practice. Tooth impaction occurs in the presence of factors that inhibit tooth eruption, such as supernumerary teeth or tumors. This is a report of permanent tooth impaction due to supernumerary teeth and pericoronal myxofibrous hyperplasia (PMH), a type of pericoronal hamartomatous lesion. An eight-year-old girl was diagnosed with an unerupted right maxillary central incisor. An inverted supernumerary tooth was present on the palatal side of the impacted central incisor, and PMH developed on the labial side of the central incisor. Interestingly, the alveolar bone on the labial side had completely disappeared. After the extraction of the supernumerary tooth and the removal of the PMH, the central incisors erupted, and the labial alveolar bone regenerated normally. Treatment for impacted teeth typically involves the removal of any existing lesions. This case is unique in that the alveolar bone of the impacted tooth regenerated following the extraction of the supernumerary tooth and removal of the PMH.

## Introduction

Tooth eruption occurs through a complex interplay of various factors, including the growth of the tooth embryo within the jawbone, root development, and jawbone growth. If these factors do not function properly, or if inhibiting factors are present, the tooth can become impacted. Less frequently, thickening of the oral mucosa and gingival hyperplasia have been reported as causes.

Supernumerary teeth, particularly those in the anterior maxilla, cause eruption failure, displacement, rotation, and median diastema of the permanent maxillary incisors [1,2]. Pericoronal myxofibrous hyperplasia (PMH), a type of pericoronal hamartomatous lesion, is characterized by odontogenic mesenchymal tissue hyperplasia with the appearance of odontogenic epithelial islands and multinucleated giant cells [3]. PMH lesions are unfavorable for eruption and are associated with impaction.

Herein, we report a case of a central incisor that had been impacted by supernumerary teeth on the palatal side of the central incisor and a PMH on the labial side of the central incisor.

## Case presentation

The female patient, aged eight years and three months (height, 123 cm; weight, 24 kg), visited our department after her local doctor noted that her maxillary right central incisor had not yet erupted and that she had a supernumerary tooth.

**Figure 1:**
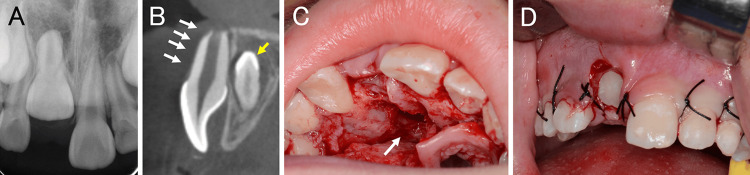
Images of the supernumerary tooth before surgery and intraoral photographs after surgery (A) Dental photographs and (B) dental cone-beam computed tomography images during the first visit. The yellow arrow indicates supernumerary teeth. The white arrow line indicates the position of the horizontal level. (C) Intraoral photographs after supernumerary tooth extraction and fenestration. The white arrow indicates an intraoral photograph of the tooth extraction socket. (D) Intraoral photo immediately after surgery.

**Figure 2:**
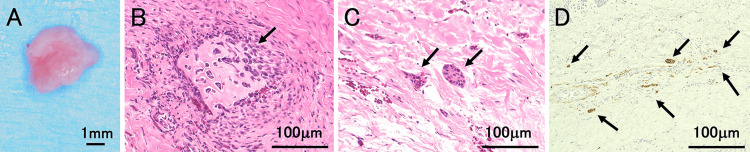
Histological findings of the surgery specimen (hematoxylin and eosin staining) (A) Extracted gingival tissue. (B) The black arrow indicates osseous tissue. (C) The black arrows indicate multiple islands of odontogenic epithelium in a basophilic myxoid stroma with collagen fibers. (D) Histological findings of the surgery specimen (immunostaining of S-100,a neuron marker). The black arrows indicate S-100-positive cells.

Intraoral findings showed that the maxillary right central incisor had not erupted, and a bulge due to supernumerary teeth was palpable in the alveolar mucosa on the palatal side. Dental and panoramic radiographic findings showed that the right upper central incisor was impacted low, and the root was incomplete (Figure 1A). Dental cone-beam computed tomography (CBCT) findings revealed an inverted, impacted supernumerary tooth on the palatal side of the right central incisor tooth germ in the maxilla (Figure 1B). An opaque image of a supernumerary tooth was observed on the palatal side of the central incisor. The tooth germ of the right central incisor moved labially, and no alveolar bone coverage was observed from the crown to the root apex (Figure 1B). The clinical diagnosis was excessively impacted teeth in the maxillary midline and delayed eruption of the right maxillary central incisor. After infiltration anesthesia, an incision was made from the distal first premolar on the right side of the maxilla to the distal side of the left canine on the maxilla, and the mucoperiosteal flap was removed. The alveolar bone corresponding to the palatal supernumerary tooth was removed, and the supernumerary tooth was extracted. Subsequently, the gingival tissue around the crown of the central incisor was removed, and fenestration was performed (Figures 1C, 1D). Gingival tissue was removed to prevent exposure of the root in the hope of regenerating the alveolar bone, and the coronal gingival was removed to allow for future traction. The surgery was performed from the incisal side. A pathological diagnosis was made on the excised gingival tissue (Figure 2A). The histopathological findings of gingival tissue revealed active bone formation (Figure 2B) and myxofibrous hyperplasia with island-like odontogenic epitheliums (Figure 2C). Furthermore, relatively thick nerve fibers (Figure 2D) were observed in the extracted gingival tissue. The histopathological diagnosis was pericoronal mesenchymal hamartoma.

**Figure 3:**
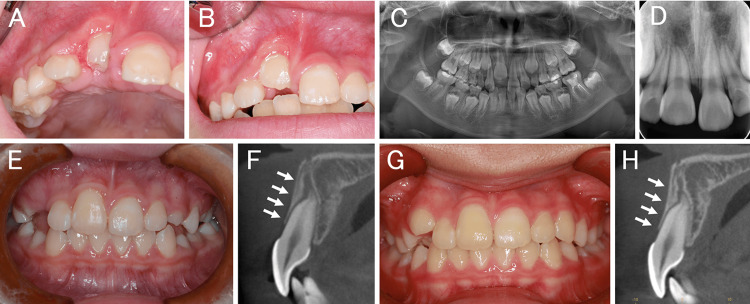
Treatment process (A) Intraoral photograph one week after surgery. (B,C) Intraoral photograph and panorama radiographic one month after surgery. (D) Dental radiographic four months after surgery. (E) Intraoral photograph and (F) cone-beam computed tomography nine months after surgery. The white arrow line indicates the position of the horizontal level. (G) Intraoral photograph and (H) cone-beam computed tomography one year and three months after surgery. The white arrow line indicates the position of the horizontal level.

Intraoral findings one week after surgery showed the crown of the central incisor (Figure 3A), and eruption of the central incisor was observed one month after surgery (Figure 3B). Panoramic radiographic taken one month after surgery and a dental radiographic taken four months after surgery are shown (Figures 3C, 3D). Nine months after surgery, the entire crown of the central incisor was visible (Figure 3E), and the alveolar bone coverage on the labial side also showed signs of recovery (Figure 3F). One year and three months after surgery, eruption of the central incisor was complete, and the labial alveolar bone coverage had also recovered (Figures 3G, 3H).

The procedure was explained to the parents of the patient, who signed an informed consent form allowing treatment procedures and publication of data.

## Discussion

Tooth impaction can be caused by systemic and local factors [4]. It is extremely rare for a disease to develop due to systemic factors, and in such cases, it often involves multiple dental impactions. It is also associated with hereditary diseases that cause bone metabolic abnormalities (such as marble bone disease and cleidocranial dysostosis), endocrine disorders (such as rickets and hypothyroidism), and syndromes (such as Down syndrome and Gardner syndrome). Local variables can be attributed to several factors. The most common cause is the presence of supernumerary teeth or odontomas in the eruption paths of the permanent teeth. Supernumerary teeth are reported to be present in 0.8% and 2.1% of primary and permanent dentitions, respectively [4,5]. They most commonly involve the anterior maxilla, followed by the mandibular premolar region [4,5]. Supernumerary teeth, particularly those in the anterior maxilla, can cause eruption failure, displacement, rotation, and median diastema of the permanent maxillary incisors. In this case, dental and panoramic radiographs showed overlapping of the maxillary right central incisor and the supernumerary tooth, suggesting that the supernumerary tooth was the cause of the impaction of the maxillary left central incisor. However, dental CBCT images taken for detailed examination not only showed that the right central incisor was deviated labially but also that the labial alveolus from the crown to the root end had no bone coverage (Figure 1B). To the best of our knowledge, there have been no reports of alveolar bone loss on the labial side of the central incisors due to supernumerary teeth on the palate of the central incisors.

PMH is characterized by hamartomatous lesions in the pericoronal tissue of teeth with delayed eruption, as proposed by Yonemochi et al. in 1998 [3]. The most common site of PMH occurrence is the mandibular first or second molar, and it has been reported to cause molar eruption failure [6]. The clinical characteristics of PMH are gingiva and alveolar ridge that appear normal and have the color of a healthy gingiva but with a slight concave depression in the part of the gingiva that covers the crown of the tooth [6]. Additionally, almost no bone tissue was observed on the crown of the tooth. PMH is characterized by an increase in myxofibrous organic components; the appearance of odontogenic epithelial components and giant cells; and the presence of accompanying bone, dentin, or cementum-like hard tissue components. In this case, no depression was observed in the gingiva covering the crown of the tooth; however, this was believed to be due to PMH occurring in contact with the labial surface of the central incisor, which has a smooth morphology. In this case, a slight concave depression of the gingiva that covered the crown of the tooth was not observed, which may be because PMH occurred in the central incisor region. Furthermore, we could not confirm the presence of PMH lesions on CBCT images. Histopathologically, PMH is transitional from the surrounding normal tissue, making it difficult to distinguish between lesions and normal sites. Therefore, because there is no capsule similar to that of benign tumors, they are difficult to diagnose using X-rays. We were unable to diagnose PMH based on clinical findings and CBCT images. The pathological diagnosis of the gingival tissue revealed myxofibrous organic components, odontogenic epithelium, giant cells, and bone-like hard tissue, which are characteristics of PMH.

In this case, we believed that there was no relationship between the supernumerary teeth and the PMH because of their relative positions. After the supernumerary tooth was extracted and the PMH was removed along with the fenestration of the central incisor, the central incisors began to erupt one month after treatment, and one year and three months later, the impacted central incisors completed the eruption into their normal positions. Furthermore, recovery of the labial alveolar bone was observed along with the eruption. Normal regeneration of alveolar bone is attributed to the removal of PMH that was inhibiting labial alveolar bone formation. The extraction of the supernumerary tooth on the palatal side created a space there, which caused the central incisors to move toward the palate, creating additional space for the alveolar bone on the labial side to regenerate.

## Conclusions

This case shows that the labial alveolar bone was lost due to a supernumerary tooth and PMH was regenerated after the extraction of the supernumerary tooth and removal of the PMH. Removal of the lesion leads to regeneration of the alveolar bone. Impaction of permanent teeth during the replacement period is a relatively common occurrence in clinical practice. If an impacted tooth caused by a supernumerary tooth is treated appropriately, natural eruption of the permanent tooth can be expected.
